# Genome sequencing, comparative analysis, and gene expression responses of cytochrome P450 genes in *Oryzias curvinotus* provide insights into environmental adaptation

**DOI:** 10.1002/ece3.11565

**Published:** 2024-06-18

**Authors:** Ming Li, Aiping Deng, Chuanmeng He, Zebin Yao, Zixuan Zhuo, Xiu yue Wang, Zhongduo Wang

**Affiliations:** ^1^ Key Laboratory of Aquaculture in South China Sea for Aquatic Economic Animal of Guangdong Higher Education Institutes Fisheries College, Guangdong Ocean University Zhanjiang China; ^2^ Guangdong Provincial Key Laboratory of Aquatic Animal Disease Control and Healthy Culture Fisheries College, Guangdong Ocean University Zhanjiang China

**Keywords:** comparative genomic analysis, cytochrome P450, expression quantification, genomics, *Oryzias curvinotus*

## Abstract

The mangrove fish (*Oryzias curvinotus*) serves as a model for researching environmental adaptation and sexual development. To further such research, we sequenced and assembled a high‐quality 842 Mb reference genome for *O. curvinotus*. Comparative genomic analysis revealed 891 expanded gene families, including significantly expanded cytochrome P450 (*CYP*) detoxification genes known to be involved in xenobiotic defense. We identified 69 *O. curvinotus CYPs* (*OcuCYPs*) across 18 families and 10 clans using multiple methods. Extensive RNA‐seq and qPCR analysis demonstrated diverse spatiotemporal expression patterns of *OcuCYPs* by developmental stage, tissue type, sex, and pollutant exposure (17β‐estradiol (E2) and testosterone (MT)). Many *OcuCYPs* exhibited sexual dimorphism in gonads, suggesting reproductive roles in steroidogenesis, while their responsiveness to model toxicants indicates their importance in environmental adaptation through enhanced detoxification. Pathway analysis highlighted expanded *CYP* genes in arachidonic acid metabolism, drug metabolism, and steroid hormone biosynthesis. This chromosome‐level genomic resource provides crucial biological insights to elucidate the functional roles of expanded *CYPs* in environmental adaptation, sexual development, early life history, and conservation in the anthropogenically impacted mangrove habitats of *O. curvinotus*. It also enables future ecotoxicology research leveraging *O. curvinotus* as a pollution sentinel species.

## Graphical abstract

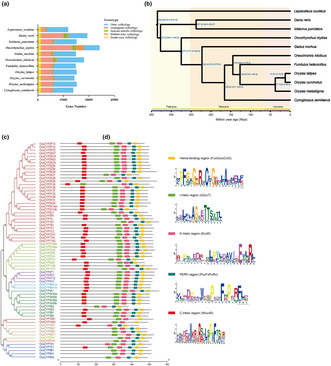

## 1 INTRODUCTION

The euryhaline *Oryzias curvinotus* inhabits mangroves across Southeast Asia, serving as an ideal research model for its hardiness and transparent embryos (Nichols & Pope, 1927). Mangroves provide optimal *O. curvinotus* habitats due to abundant resources supporting growth and reproduction (Wu et al., 2018; Xu et al., 2010). However, mangrove damage from pollution creates survival challenges for *O. curvinotus* (Morse et al., 2007). As sensitive indicators, *O. curvinotus* are used in monitoring programs to quantify biological impacts of contamination and inform conservation (Henczová et al., 2006; Simon et al., 1997).

Environmental estrogens (EEs), among the first reported environmental endocrine disruptors, are firmly established as interfering agents that adversely impact the endocrine system of organisms (Younes, 1999). Originating primarily from the discharge of industrial pollutants, an escalating concentration of environmental endocrine is being detected in lakes, estuaries, and marine habitats (Goksøyr, 2006; Gross‐Sorokin et al., 2006; Noppe et al., 2007). These contaminants disrupt hormone synthesis and metabolism to interfere with the growth and reproduction of aquatic species (Lau Wong, 1991). Therefore, this study selects E2 and MT as endocrine disruptors.

Intriguingly, some male *O. curvinotus* lack the sex gene *dmy*, differing from prior findings (Dong et al., 2021; Matsuda et al., 2003). This discovery makes *O. curvinotus* a valuable model to elucidate sex determination mechanisms. Meanwhile, invasive *Gambusia affinis* threatens *O. curvinotus* through competition and predation following introduction for mosquito control (Xiao et al., 2020; Yan et al., 2009). Given declining populations, understanding *O. curvinotus* biology is urgent for conservation.

Cytochrome P450 (*CYP*) enzymes serve as reliable biomarkers to assess biological impacts of aquatic pollutants (Sabbioni et al., 2006). As an ancient, ubiquitous superfamily, *CYPs* catalyze diverse reactions involving both endogenous and exogenous compounds (Mansuy, 1998). In particular, *CYP1A* is highly sensitive to pollutants, getting significantly induced to metabolize toxins (Alqahtani et al., 2023; Goks, 1995). Multiple scholarly inquiries have delved into the intricate impacts of environmental pollutants on the *CYPs* within various organisms, unveiling the complex interplay between these contaminants and biological systems. Lacy et al. meticulously examined the consequences of a synergistic exposure to elevated temperatures and insecticides on the swimming patterns and hepatic *CYPs* expression profiles in goldfish, underscoring the latent perils posed by environmental pollutants to the behavioral and physiological integrity of fish species (Lacy et al., 2023). Concurrently, Berenbaum et al. provided a comprehensive overview of the mechanisms employed by insects to combat plant secondary metabolites through their *CYP* system, emphasizing the pivotal role of this enzymatic machinery in insect‐plant interactions (Berenbaum et al., 2021). Furthermore, studies conducted by Georgiades et al. and Kilemade et al. on aquatic organisms such as sea stars and lampreys revealed similar susceptibilities to environmental pollutants, particularly perturbations in their *CYP* systems (Georgiades et al., 2006; Kilemade et al., 2009). Mammalian *CYPs*, a crucial component of the ecological web, is also susceptible to the influence of environmental pollutants. Segura‐Aguilar et al. demonstrated that organic halogen pollutants may perturb estradiol metabolism in rats, potentially contributing to the development of estrogen‐dependent malignancies (Segura‐Aguilar et al., 1997). Additionally, Van Der Weiden et al. and Roos et al. elucidated the induction patterns of *CYP* in fish and minipigs, respectively, following exposure to contaminated sediments and PAH‐laced soil, thereby affirming the pervasive effects of environmental pollutants on *CYPs* across diverse organisms (Roos et al., 2002; Van Der Weiden et al., 1993). Numerous studies reveal *CYP1A*'s key role in fish xenobiotic metabolism and utility to monitor contamination (Rhee et al., 2013; Whyte et al., 2000). Beyond biomarker applications, some *CYPs* influence sex determination by regulating steroid synthesis. *CYP19* (aromatase) catalyzes estrogen production (Simpson et al., 1994), while *CYP17*, *CYP51*, *CYP11*, and *CYP21* affect estrogen/androgen metabolism (Yu et al., 2003). Other *CYPs* like *CYP26* disrupt retinoic acid pathways, impacting ovary function and meiosis initiation (Hernandez et al., 2007; Le Bouffant et al., 2010). Exploring diverse *CYP* family members is elucidating their complex functions in detoxification, development, and reproductive physiology in fish.

This study generated a high‐quality 842 Mb reference genome using integrated sequencing approaches. Comparative genomics with other fish revealed expanded gene families, notably cytochrome P450s (*CYPs*), suggesting adaptation to mangroves. We identified 69 *CYPs* and characterized diverse expression patterns developmentally, by tissue, sex, and after pollutant exposures. This genome provides crucial resources to elucidate *CYP* roles in environmental adaptation, sexual differentiation, and conservation in *O. curvinotus*. The evolutionary perspective gained on expanded detoxification genes also informs research leveraging this species as a sentinel to monitor anthropogenic threats to fragile mangrove ecosystems.

## 2 METHODS

### 2.1 Sample collection and sequencing

Fish were collected from the Gaoqiao Mangrove Nature Reserve in Zhanjiang City, Guangdong Province, China. High‐molecular weight genomic DNA was extracted using TIANamp Marine Animal DNA Kits (TIANGEN, Beijing, China) and quantified by agarose gel electrophoresis and spectrophotometry with a NanoDrop 2000 (Thermo Scientific, USA).

We generated sequencing data using four approaches: PacBio (Pacific Biosciences, USA) long reads, 10X Genomics linked reads, Hi‐C scaffolding, and Illumina short reads. Illumina libraries were constructed with 350 bp fragments and sequenced on a HiSeq PE150 platform (Illumina, USA). The 10X Genomics (Pleasanton, CA, USA) and PacBio libraries used 50 kb and 20 kb inserts, respectively. Hi‐C libraries were prepared using standard protocols (Rao et al., 2014). All libraries were sequenced on Illumina or PacBio platforms per manufacturer instructions. This multi‐technology sequencing produced over 379 Gb of genomic data.

The embryos were collected from the fertilized eggs of the domesticated offspring of the *O. curvinotus* population in the Gaoqiao Mangrove Nature Reserve, Zhanjiang City, Guangdong Province, China. The parents were separated the day before the experiment according to a male‐to‐female ratio of 3:2 and mixed at 8 am the next day to allow spawning and fertilization within 1 hour. The fertilized eggs were collected, the egg‐binding filaments were removed, and the eggs were placed in a petri dish for cultivation in freshwater (0 ppt). The room temperature was controlled at 26 ± 0.5°C, with a light–dark cycle ratio of 14 h:10 h. Embryonic developmental stages were observed under a stereomicroscope, and embryos at specific developmental stages were selected, including the embryonic disc formation stage, morula stage, early gastrula stage, mid‐gastrula stage, later gastrula stage, neurula stage, 10‐somites stage, nine‐somites stage, 16‐somites stage, the eyed stage, notochord vacuolization completed stage, spleen development stage, and hatching stage. In addition, adult fish tissues were also collected. The brain, liver, gills, gonads, muscles, and eyes were dissected and collected. Additionally, the gonads and brains of female, male, and male lacking *dmy* were also dissected and collected. The biological replication number was 3.

17β‐estradiol (E2) and testosterone (MT) (Sigma, USA) were dissolved in anhydrous ethanol solvent (Sularbio, Beijing) to prepare a stock solution of 20,000 μg/L each. Fifty *O. curvinotus* juveniles within 6 h after hatching were randomly selected and placed in a 1 L glass culture dish. To each experimental group, 100 μL of the respective E2 and MT solution was added, while the control group was 1/5000 of the anhydrous ethanol solvent. Experiments were repeated three times. The final concentration of E2 and MT in the experimental groups was 2 μg/L. The juveniles were incubated in an environment maintained at 26 ± 0.5°C, with a 14‐hour light cycle and 10‐hour dark cycle, and the salinity remained constant at 0 ppt throughout the duration of the experiment. Total RNA was extracted from the above‐mentioned tissues using the TRIzol method. The integrity and quality of RNA were detected using the same methods as for DNA detection. High‐quality RNA was used to construct a cDNA library using the Illumina TruSeq RNA kit. Transcriptome sequencing was performed on the Illumina HiSeq PE150 sequencing platform. The genomic reads are accessible at NCBI PRJNA821560, albeit the project was exclusively designed for investigating population genetic diversity and was not originally intended for the purpose of genome assembly.

### 2.2 Genome survey and de novo assembly

Raw Illumina reads were filtered, with adapters, low quality (<5) bases over 20% and ambiguous (N) bases over 10% removed using Trimmomatic (version 0.39) (Bolger et al., 2014). 17‐mer spectrum were selected for counts using Jellyfish (version 2.3.0) (Marçais & Kingsford, 2011) to estimate genome size and other features, set ‐m to 17. PacBio long reads were self‐corrected and assembled into contigs with Falcon (version 3.1.3) (Pendleton et al., 2015), then polished using Quiver (Chin et al., 2013). The 10X Genomics linked reads were combined with the PacBio contigs using fragScaff (version 140324.1) (Adey et al., 2014) and gaps filled by Pilon (version 1.24) (Walker et al., 2014). Hi‐C data were used to cluster, sort, and orient contigs into chromosome‐length scaffolds with Juicer (version 2.20.00) set ‐s to Hind III (Durand et al., 2016), 3D‐DNA (version 180114) set ‐r to 2 (Dudchenko et al., 2017), and Juicebox (version 1.11.08) (Robinson et al., 2018).

The genome assembly was assessed using multiple quality metrics. Reads were aligned with BWA (version 0.7.17) (Li & Durbin, 2009) to calculate coverage and variation using SAMtools (version 1.17) (Li, 2011). CEGMA (version 2.5) (Parra et al., 2007) and BUSCO (version 5.4.7) (Simão et al., 2015) assessed gene content completeness. SNP homozygosity, read coverage, and conserved gene metrics demonstrated the high continuity, accuracy and completeness of this chromosome‐level genome assembly.

### 2.3 Genome annotation

Repetitive elements were identified using RepeatMasker (version 4.1.5) set ‐xsmall ‐nolow ‐no_is ‐norna (Tarailo‐Graovac & Chen, 2009) with RepBase (version 20181026) (Jurka et al., 2005) and by merging de novo libraries from RepeatModeler (version 2.0.1) (Flynn et al., 2020) and LTR‐FINDER (version 1.07) set ‐D 15000 ‐d 1000 ‐L 7000 ‐l 100 ‐p 20 ‐C ‐M 0.85 (Xu & Wang, 2007). Non‐redundant repeats were compiled.

Protein‐coding genes were predicted by Augustus (version 3.3.2) (Stanke et al., 2006), SNAP (version 2013_11_29) (Leskovec & Sosič, 2017), and GlimmerHMM (version 3.0.4) (Majoros et al., 2004) on the repeat‐masked genome. RNA‐seq data was aligned with TopHat (version 2.1.1) (Trapnell et al., 2009) and assembled into gene models with Cufflinks (version 2.2.1) (Trapnell et al., 2012). Models were integrated using EvidenceModeler (EVM) (version 2.1.0) (Haas et al., 2008) and refined by PASA (version 2.5.3) (Haas, 2003) to add UTRs and alternative splicing.

Functional annotation of genes was performed by searching the SwissProt (Boeckmann, 2003), Non‐ redundant (Nr, from NCBI) and Kyoto Encyclopedia of Genes and Genomes (KEGG) (Kanehisa et al., 2012) databases using BLASTP (version 2.2.31) set ‐e to 1e‐5 (Camacho et al., 2009) and comparing with the Gene Ontology (GO) database (Ashburner et al., 2000) using Blast2GO (version 6.0) set ‐e to 1e‐5 (Conesa et al., 2005).

Non‐coding RNAs were identified by scanning with tRNAscan‐SE (version 2.0.12) set ‐E ‐I (Chan & Lowe, 2019), RNAmmer (versino 1.2) (Lagesen et al., 2007), and Infernal (Nawrocki & Eddy, 2013) against Rfam (Kalvari et al., 2018) to find tRNAs, rRNAs, miRNAs, and snRNAs.

### 2.4 Gene family analysis

Genome data for 10 other teleosts was obtained from Ensembl (Braasch et al., 2016; Chen, 2014; Conte, 2017; Gao et al., 2021; Howe et al., 2013; Johnson et al., 2019; Kasahara et al., 2007; Kim et al., 2018; Kirubakaran et al., 2020; Liu et al., 2016). Orthologous gene families were classified by BLAST (version 2.2.31) set ‐e to 1e‐5 (AltschuP et al., 1990) against *O. curvinotus* proteins using OrthoFinder (version 2.5.5) set ‐M msa ‐S diamond ‐T fasttree (Emms & Kelly, 2019).

Single‐copy orthologs were aligned with MAFFT (version 7.520) (Katoh & Standley, 2013) and used to construct a maximum likelihood phylogeny with IQ‐TREE (Minh et al., 2020), calibrated with two divergence times from TimeTree (Yang, 2007).

Gene family expansions and contractions were estimated with CAFÉ (version 5.10) (Mendes et al., 2021) using a *p*‐value threshold of 0.05. Expanded families were tested for GO and KEGG enrichment with clusterProfiler (version 4.2.2) (Yu et al., 2012).

### 2.5 Cytochrome P450 gene family (*CYP*) annotation and analysis

*CYP* genes were manually annotated to ensure accuracy. The P450 HMM PF00067 model (Mistry et al., 2021) was searched against the genome using hmmer (version 3.3.2) (Eddy, 2009). Putative *CYPs* were identified by BLAST (version 2.2.31) against other teleost *CYPs* (Nelson, 2009). Gene structures were determined by transcriptome alignment and conserved domains confirmed using CDD (Lu et al., 2020). Incomplete genes were corrected with FGENESH. Translated sequences were queried with hmmer to compile the final *OcuCYPs* list and named per convention (Nelson, 2009).

Phylogenetic trees were constructed by aligning *O. curvinotus*, zebrafish, and marine medaka *CYP* protein sequences using MEGA11 (Kumar et al., 2016) and iTOL (https://itol.embl.de/). Chromosomal localization was performed with TBtools (version 2.085) (Chen et al., 2010). Multiple alignments identified conserved motifs with MEME (https://meme‐suite.org/meme/) (Bailey et al., 2006), also visualized in TBtools.

### 2.6 Expression profiling and qPCR validation of *OcuCYP*s

RNA‐seq data were analyzed to investigate *OcuCYPs* expression patterns across four sample types: (1) developmental stages, (2) tissues, (3) gonads/brains by sex, and (4) larvae exposed to chemicals. Reads were mapped to the genome with HISAT2 (Kim et al., 2015) and counted with FeatureCounts (Liao et al., 2014). TPMs were calculated and expression profiles visualized using TBtools. Differential expression analysis was performed with DESeq2 (Love et al., 2014).

Fourteen *OcuCYPs* were validated by qPCR across adult fish's tissues (brain, liver, gill, gonad, muscle, and eye) and female, male (gonad and brain) using gene‐specific primers (Table S7) and rps4x as a reference (*n* = 3). Reactions were performed using PerfectStart Green qPCR SuperMix kit (TransGen Biotech) on a Bio‐Rad real‐time PCR system per manufacturer protocols. Relative expression was calculated by the $2^{-\Delta \Delta C_{t}}$ method. Calculating in Excel, Δ*C*_t_ = cq value of the reference gene – cq value of the target gene. The average Δ*C*_t_ is then taken. ΔΔ*C*_t_ = each individual Δ*C*_t_ − average Δ*C*_t_. Finally, the relative expression level  = $2^{-\Delta \Delta C_{t}}$ GraphPad Prism was used for visualization.

## 3 RESULTS

### 3.1 Raw data

Using a combination of sequencing approaches, we generated 379.72 Gb of genomic data. This included 103.5 Gb of Illumina reads (110X coverage), 117.64 Gb of 10X Genomics reads (125X coverage), 82.6 Gb of Hi‐C reads (88X coverage), and 75.98 Gb of PacBio reads (81X coverage). Full sequencing statistics are provided in Table S1. The aggregate high‐coverage data from multiple technologies enabled the assembly of a high‐quality chromosome‐level reference genome.

### 3.2 Genome assembly and annotation

**FIGURE 1:**
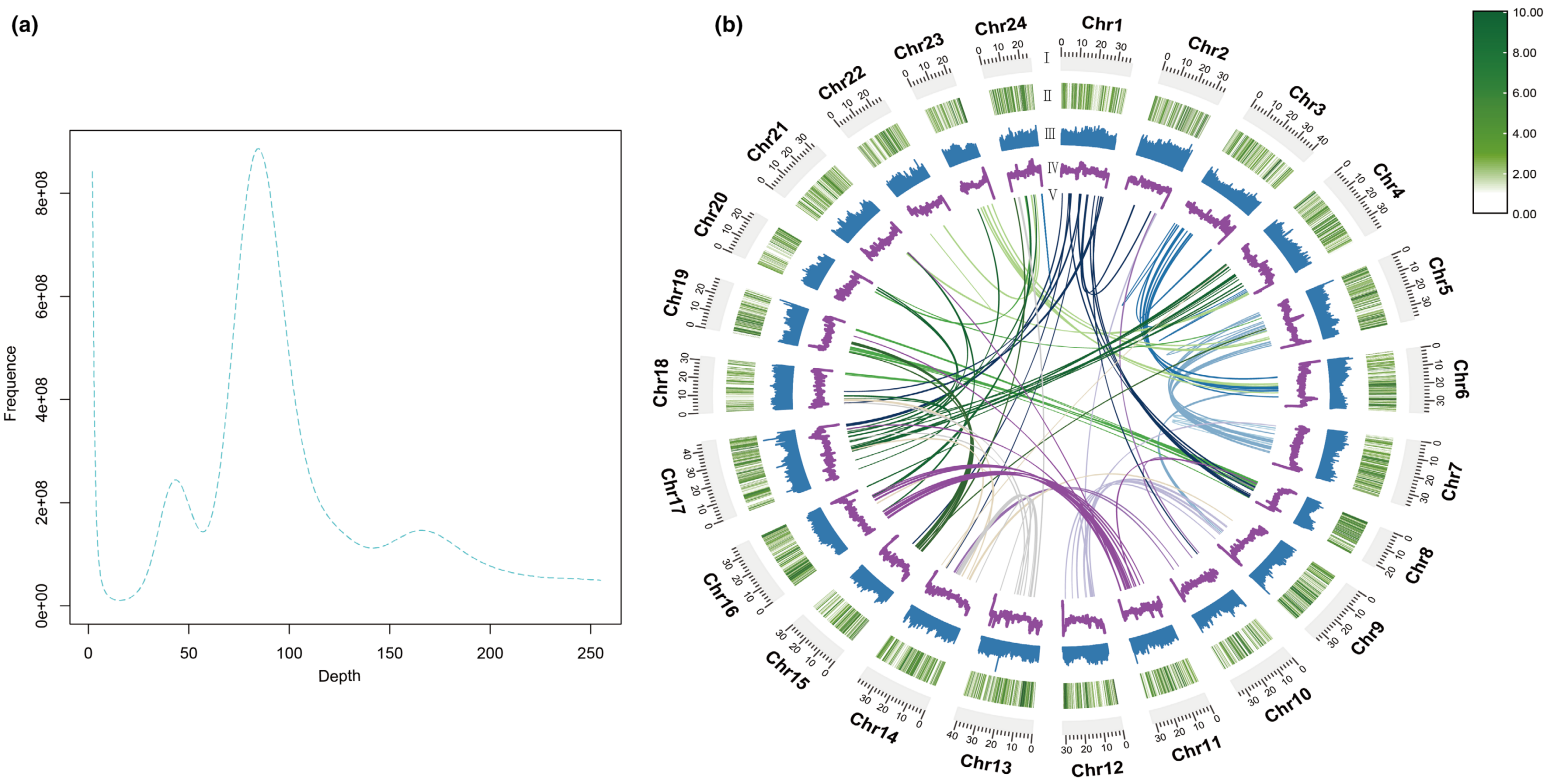
(a) 17‐mer depth distribution for genome survey and estimation of genome features. (b) Chromosome‐level genome assembly of *O. curvinotus*. From outside to inside circles: (I) 24 chromosomes (unit Mb); (II) gene density on each chromosome; (III) repeat sequence density; (IV) genomic GC content; (V) genomic collinearity.

K‐mer analysis estimated the genome size at ~948 Mb with 47% repeats (Table S2). There is a main peak of depth at around 90× when Kmer = 17 (Figure 1a). De novo assembly yielded 867 Mb contigs (N50 = 1.37 Mb) and 872 Mb scaffolds (N50 = 34.5 Mb) (Table S3). Integration of Hi‐C data produced an 842 Mb, 24‐chromosome genome (Figure 1b). Quality assessments showed high continuity (93.45% read mapping) and completeness (96% of conserved genes present) (Tables S4 and S5). The BUSCO assessment indicates that the genome has a high level of completeness at 97.6%, with 94.8% being complete and single‐copy BUSCOs (S), 2.8% being complete and duplicated BUSCOs (D), and only 0.7% fragmented BUSCOs (F) and 1.7% missing BUSCOs (M) (Table S9).

The genome is 40.5% repetitive, primarily unclassified, DNA, and LINE elements (Table S6). A total of 22,409 protein‐coding genes were annotated, with 91.7% supported by homology evidence. Most genes had functional descriptions from SwissProt (85.4%), NR (91.4%), KEGG (91.2%), and GO (59.2%). Thousands of non‐coding RNAs were also identified, including tRNAs, rRNAs, miRNAs, and snRNAs.

### 3.3 Gene family clustering and phylogenetic analysis

**FIGURE 2:**
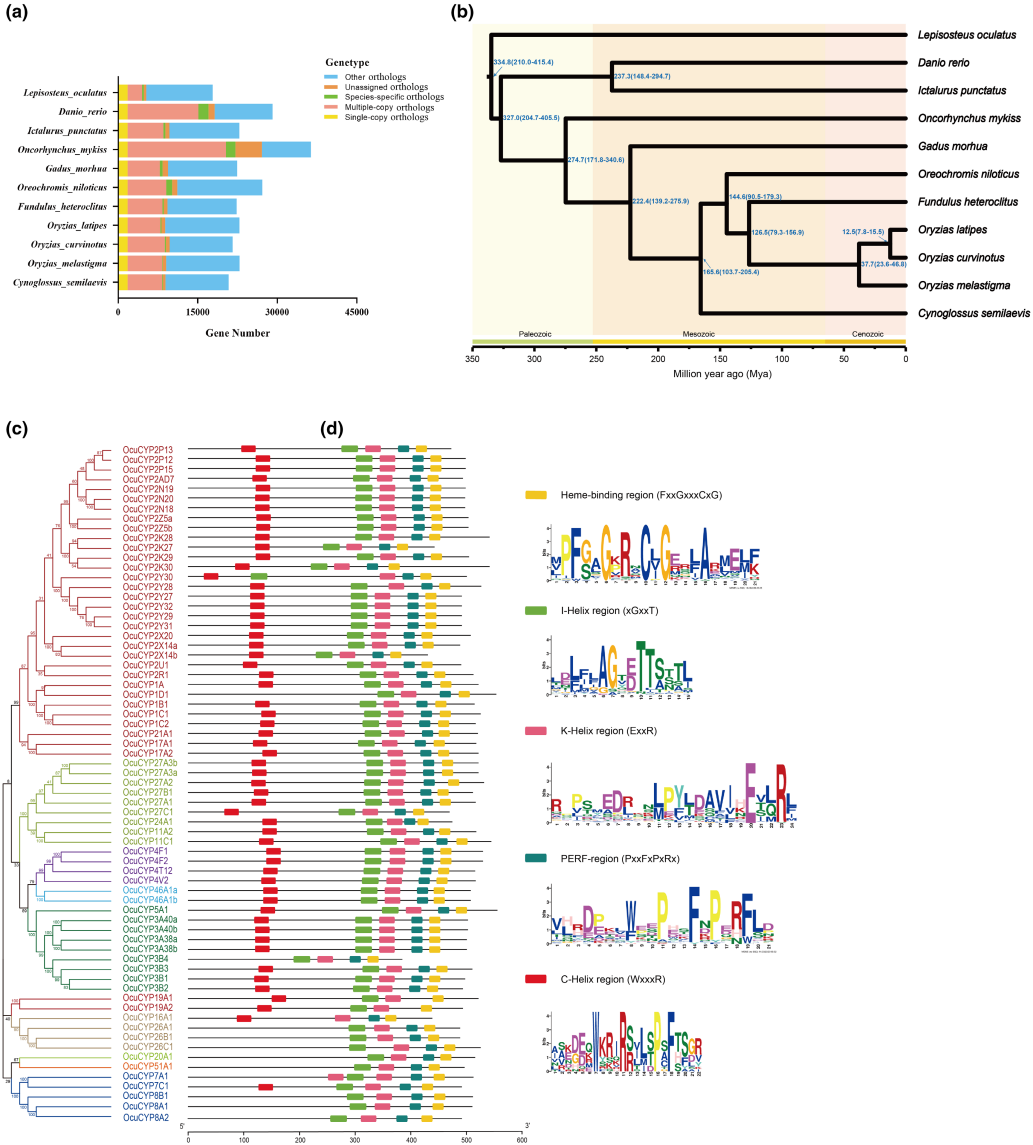
(a) Orthologous gene family clusters across 11 teleost genomes compared to *O. curvinotus*. Whole genome data were obtained from the Ensembl database (http://asia.ensembl.org/index.html) for another 10 teleost species, including *Cynoglossus semilaevis* (GCA_000523025.1), *Danio rerio* (GCA_000002035.4), *Fundulus heteroclitus* (GCA_000826765.1), *Gadus morhua* (GCA_902167405.1), *Ictalurus punctatus* (GCA_001660625.1), *Lepisosteus oculatus* (GCA_000242695.1), *Oncorhynchus mykiss* (GCA_013265735.3), *Oreochromis niloticus* (GCA_001858045.3), *Oryzias latipes* (GCA_002234715.1), and *Oryzias melastigma* (GCA_002922805.1). (b) Maximum likelihood phylogenetic tree and divergence time estimation for *O. curvinotus* and 10 other teleosts. Database TimeTree (http://www.timetree.org/) was selected as the calibration source for the following divergence times: (1) *Lepisosteus oculatus* and *Oryzias latipes* (298.8–342.5 Mya), (2) *Oreochromis niloticus* and *Fundulus heteroclitus* (83.0–103.8 Mya). (c and d)Phylogenetic relationship and conserved motifs of *OcuCYPs* proteins. (c) Phylogenetic tree of *OcuCYPs*, with different colors indicating the *CYPs* that make up a clan. (d) Distribution of five conserved motifs on *OcuCYPs* proteins, with squares of five colors representing the regions of the five motifs: yellow for the heme‐binding region, green for helix I, pink for helix K, turquoise for the PERF region, and red for helix C.

Gene family clustering showed that protein‐coding genes of all species were clustered into 20,953 orthogroups, of which 1784 single‐copy genes were identified. There were 207 Species‐specific genes and 7004 multiple‐copy genes, and the clustering of genomic gene families was similar to that of the closely related *Oryzias* species (Figure 2a).

Phylogenetic analysis based on 1784 single‐copy orthologs revealed *O. curvinotus* formed a monophyletic clade with other *Oryzias* genus members, sharing proximity with *Fundulus heteroclitus* and *Oreochromis niloticus* (Figure 2b). Divergence time estimation dated the *O. curvinotus–O. latipes* speciation event at ~12.5 million years ago.

Analysis of conserved motifs identified five key *CYP* enzyme motifs, with 55 *OcuCYPs* (79.7%) possessing all 5 motifs (Figure 2c–e). The heme‐binding and helix K motifs were present in all *OcuCYPs*. However, PERF, Helix I, and Helix C motifs were absent from some proteins. Helix C was the most frequently absent, missing in seven *OcuCYPs*.

### 3.4 Expansion and contraction of gene family

**FIGURE 3:**
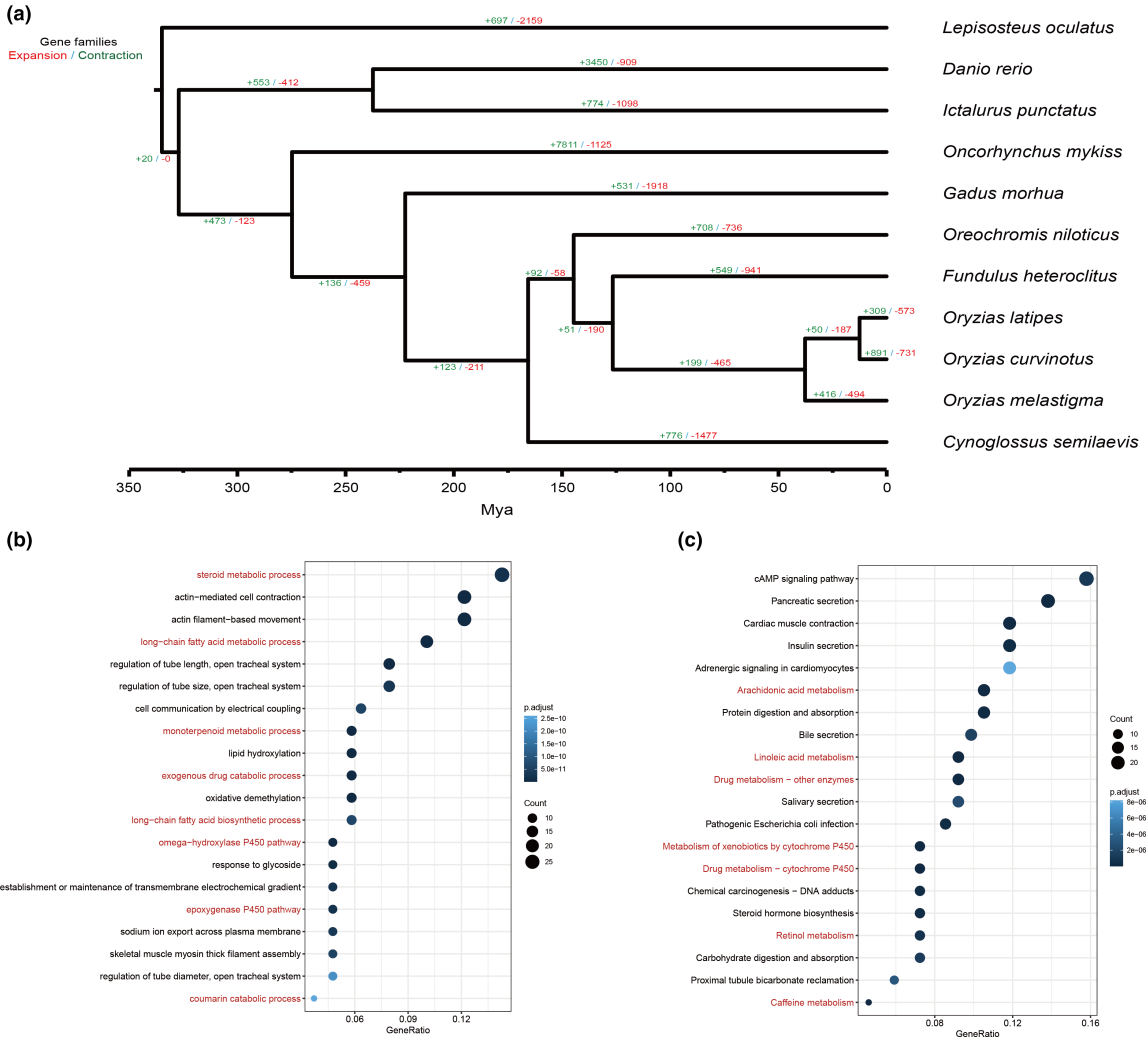
(a) Expansions (red) and contractions (green) of gene families in *O. curvinotus* genome compared to other teleosts. (b) GO enrichment analysis of expanded gene families in *O. curvinotus*. (c) KEGG enrichment analysis of expanded gene families in *O. curvinotus*. Use *λ* parameter to calculate birth rate and death rate with *p* ≤ .05 as the significance threshold.

Comparative genomics revealed 891 expanded and 731 contracted gene families in *O. curvinotus* (Figure 3a). Among these, 107 families showed significant expansion and 293 significant contraction (*p* < .05).

Expanded families were enriched for 981 GO terms and 57 KEGG pathways related to metabolism (Figure 3b,c), including steroid metabolism (GO:0008202) and arachidonic acid metabolism (ko00590). Analysis of expanded genes and pathways indicated enrichment for cytochrome P450 (*CYP*) genes. In total, 16 significantly expanded *CYP* genes were annotated, concentrated in Clans 2 and 4.

Contracted families were enriched in pathways including neuroactive ligand‐receptor interaction (ko04080) and G protein‐coupled receptor activity (GO:0004930).

### 3.5 Genome‐wide identification of the P450 gene family in *O. curvinotus*

Manual annotation identified 69 cytochrome P450 (*CYP*) genes in *O. curvinotus* (*OcuCYPs*), spanning 10 clans and 18 families. The *CYP2* family was the largest, with 24 members.

The 69 *OcuCYPs* encoded proteins ranging from 384 to 555 amino acids in length and 44.16 to 63.79 kDa in predicted molecular weight (Table S8). Theoretical isoelectric points spanned 5.39 to 9.62, with 53 *OcuCYPs* (76.81%) exhibiting alkaline isoelectric points over 7.

Specific details on the structural features of the proteins encoded by each of the 69 annotated *OcuCYPs* are provided in Table S8.

### 3.6 Chromosome location and homology analysis of *OcuCYPs*

**FIGURE 4:**
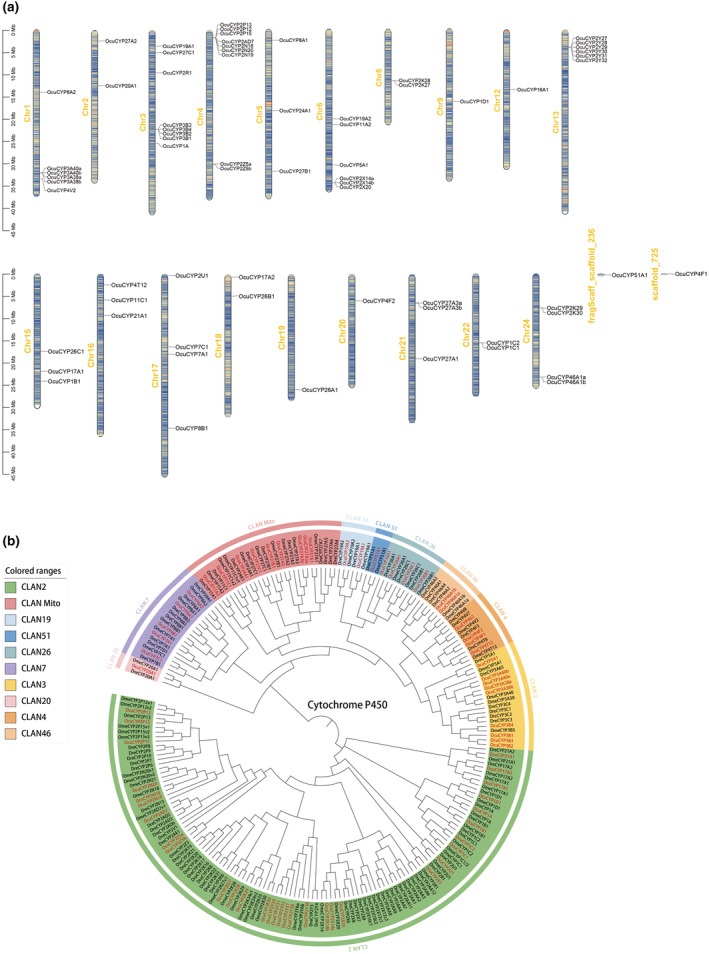
(a) Chromosomal localization of *OcuCYP* genes across the *O. curvinotus* genome. (b) Phylogenetic tree comparing *OcuCYPs* (red) to *CYPs* from other model fish species.

*OcuCYPs* were localized across 19 chromosomes and 2 scaffolds, distributed unevenly with most at chromosome ends (Figure 4a). Chr4 contained the most *OcuCYPs* (8 genes) while Chr9, Chr12, Chr19, and Chr20 had the fewest (1 gene each). Numerous *OcuCYPs* showed tandem duplications, including 2–3 repeats in the *CYP2*, *CYP3*, *CYP27*, and *CYP46* families.

Phylogenetic analysis revealed *O. curvinotus CYPs* clustered with those from zebrafish and marine medaka, as expected (Figure 4b). The *CYP2* family represented the largest expansive clan, though some subfamilies like *CYP2R/U* were highly conserved. While zebrafish had more *CYP* genes overall, *O. curvinotus* uniquely possessed *CYP16A1* yet lacked *CYP39A1* and *CYP2AA/3C* subfamilies present in other species.

### 3.7 Early developmental and tissue‐specific expression of *OcuCYPs*

**FIGURE 5:**
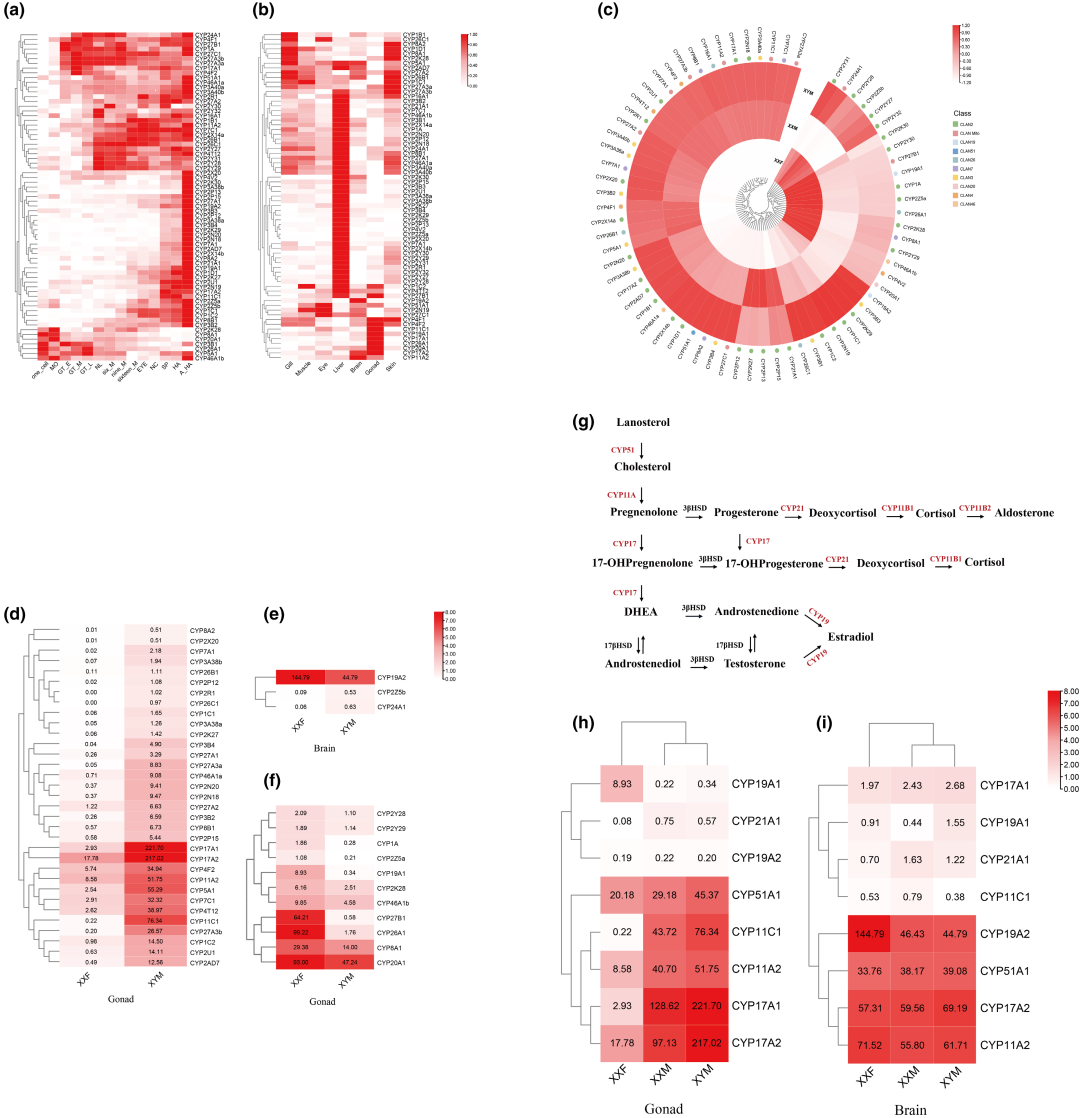
(a, b) Developmental stage and tissue‐specific expression profiles of *OcuCYPs* based on RNA‐seq data. (a) Heatmap of *OcuCYPs* expression in 14 developmental stages from embryonic development to post‐emergence: one‐cell (blastodise stage), MO (morula stage), GT_E (early gastrula stage), GT_M (mid‐gastrula stage), GT_L (later gastrula stage), NL (neurula stage), six_M (6 somites stage), nine_M (9 somites stage), sixteen_M (16 somites stage), EYE (the eyed stage), NC (complete vacuolation of the notochord stage), SP (spleen development stage), HA (hatching stage), and A_HA (hatched larvae). (b) Heatmap of *OcuCYPs* expression in seven tissues. (c) Heatmap of *OcuCYP* expression in gonads of male and female *O. curvinotus*. (d, e, and f) Differentially expressed *OcuCYPs* between male and female tissues of *O. curvinotus*. (g, h, and i) *CYPs* involved in steroidogenesis and their expression patterns in *O. curvinotus* tissues.

RNA‐seq analysis revealed varied *OcuCYP* expression patterns during *O. curvinotus* early development (Figure 5a). Most *OcuCYPs* were highly expressed in larval stages post‐hatching, though some like *CYP2K28* were elevated earlier. Four expression profiles were observed: (1) continuous from pre‐cell division, for example *CYP1A*; (2) upregulated from neurulation onwards, for example *CYP11A2*; (3) higher in late embryogenesis, for example *CYP19A1/2*; (4) elevated in early blastula stage.

Tissue‐specific analysis showed predominantly liver‐enriched expression for many *OcuCYPs* (Figure 5b). Of 32 Clan 2 members, 24 had peak liver expression. Numerous CYPs were also abundant in gills and skin. Select genes like *CYP17A1/2* and *CYP19A1* exhibited elevated gonad expression.

### 3.8 Sexual dimorphism expression of the *OcuCYPs*

RNA‐seq analysis of gonads showed largely consistent *CYP* expression in XY and XX testes, but differences between ovaries and testes (Figure 5c). Differential screening revealed 44 *OcuCYPs* with significant sex‐biased expression in gonads (Figure 5d,f). In contrast, only 3 *CYPs* differed between male and female brains, including gonad‐enriched *CYP19A2* with female‐dominant brain expression (Figure 5e).

Schematics illustrated 6 *CYP* families involved in steroidogenesis (Figure 5g). In gonads, *CYP17A1/A2* and *CYP11A2/C1* were testis‐elevated while *CYP19A1* was ovary‐enriched (Figure 5h,i). In brains, most steroidogenic *CYPs* had low expression, except *CYP19A2* with female‐biased expression.

### 3.9 Expression of *OcuCYPs* under E2 and MT exposure

**FIGURE 6:**
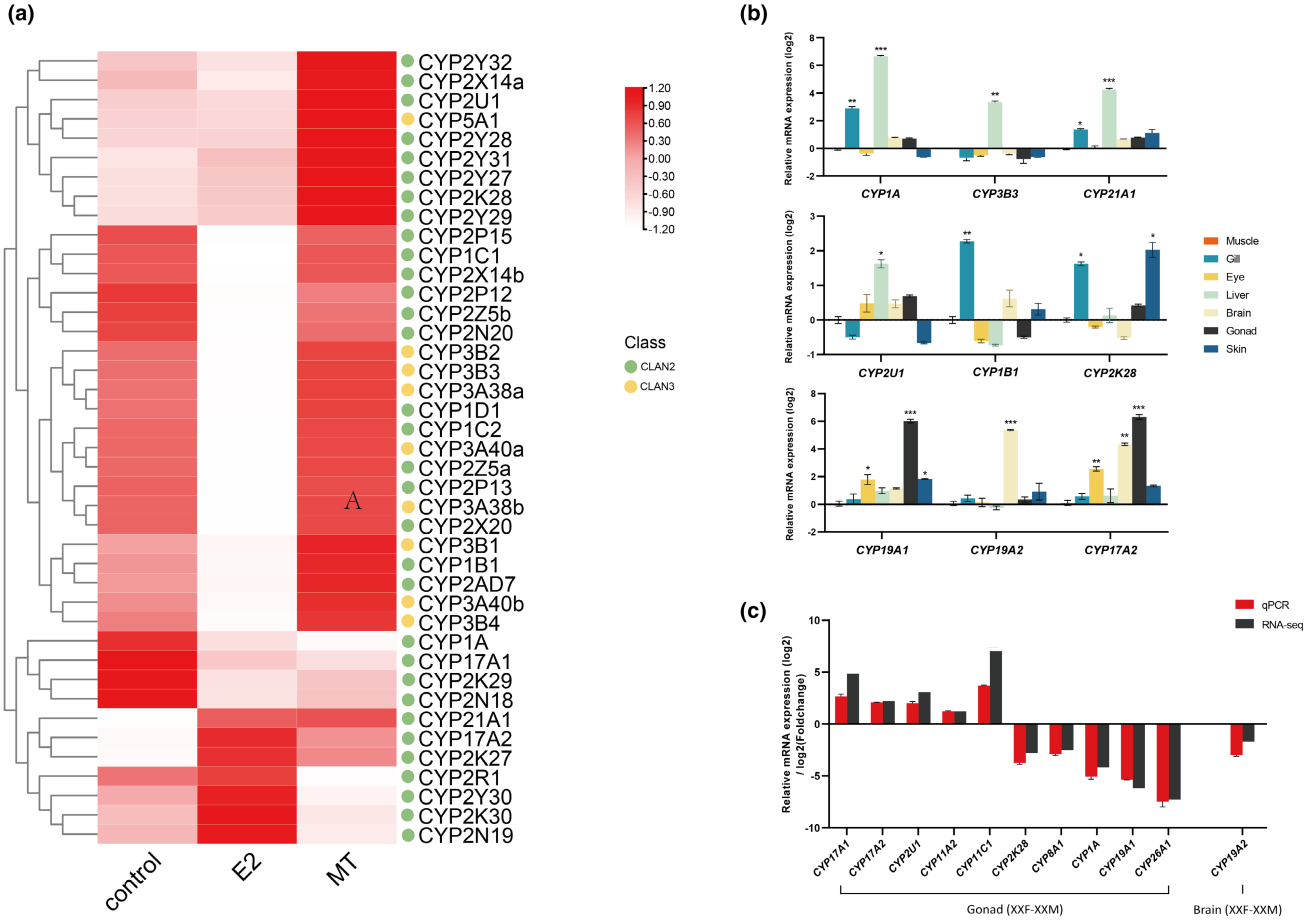
(a) Expression changes of detoxification‐related *OcuCYPs* after chemical exposures. (b, c) qPCR validation in tissues of *O. curvinotus*. (b) The abscissa of the axis represents the gene, and the ordinate represents the log_2_ value of the relative expression, normalized to the average relative expression of muscle tissue (*n* = 3). Significant differences (*p* < .05, *p* < .01, and *p* < .001) are denoted by asterisks (*). (c) The abscissa of the coordinate axis represents the gene, and the ordinate represents the log_2_ value of the relative gene expression in qPCR and the log_2_ fold change of the gene in RNA‐seq, relative to the average relative expression of the gene in the female gonad and brain(*n* = 3). Relative expression was calculated by the $2^{-\Delta \Delta C_{t}}$ method.

Due to the sensitivity of cytochrome P450 genes to environmental pollutants, Clan 2 and Clan 3 members can serve as biomarkers indicating exposure. RNA‐seq of *O. curvinotus* larvae exposed to model chemicals E2 and MT revealed significant expression changes in most P450s, including 11 Clan 2 genes typified by *CYP1A* (Figure 6a). Their altered regulation under both exposures confirms the responsiveness of expanded *OcuCYPs* to exogenous compounds, likely reflecting their roles in metabolism and detoxification.

### 3.10 qPCR validation of RNA‐seq data

qPCR validation of 14 *OcuCYPs* largely confirmed the RNA‐seq expression patterns (Figure 6a,b). In tissues, *CYP1A*, *CYP3B3*, *CYP21A1*, and *CYP2U1* were liver‐enriched, *CYP1B1* was gill‐elevated, and *CYP2K2*8 was higher in gill and skin (Figure 6b). Gonad‐associated *CYP19A1/2* and *CYP17A2* also showed gonad and brain specificity. In gonads, *CYP17A1/2*, *CYP2U1*, and *CYP11A2/C1* were testis‐biased while *CYP2K28*, *CYP8A1*, *CYP1A*, *CYP19A1*, and *CYP26A1* were ovary‐elevated (Figure 6c). *CYP19A2* displayed female‐dominant expression in brains (Figure 6c).

## 4 DISCUSSION

Genomics serves as a crucial tool for understanding biological adaptability, evolution, and ecology, with profound implications for the protection and utilization of biodiversity (Bernardi, 2005; Kelley et al., 2016; Shao et al., 2017; Star et al., 2011; Wang et al., 2015; Yang et al., 2019; Zhu et al., 2021). Especially when confronted with the challenges of global environmental change and biodiversity loss, in‐depth research on specific species becomes particularly significant (Meza‐Joya et al., 2023). In this study, we focused on *O. curvinotus*, a fish species that lives in the complex and diverse mangrove waters (Wu et al., 2018). Mangrove ecosystems play an indispensable role in global carbon cycling, coastal line protection, and biodiversity maintenance, yet they are also among the ecosystems most threatened by pollution and habitat destruction (Rahmadi et al., 2023; Song et al., 2023). The findings of this study not only enhance our understanding of the genomic structure and function of *O. curvinotus* but also provide molecular insights into the protection and sustainable management of mangrove ecosystems. Through further research on these gene families, we can gain a deeper understanding of how *O. curvinotus* survives and reproduces in constantly changing environments, which holds significant guidance for the protection of global mangrove ecosystems. Furthermore, these discoveries also offer a new perspective on exploring the adaptation mechanisms of other organisms in similar environments, contributing to the advancement of biodiversity conservation and ecological research.

The choice of *O. curvinotus* as the research subject is not only due to its unique ecological niche and sensitivity to environmental changes. The high‐continuity 842 Mb *O. curvinotus* genome provides a valuable resource for genetic research on this species (Figure 1b, Tables S4, S5, and S9). Its quality metrics, including 93% read mapping and 99% coverage, indicate good accuracy for studies of environmental adaptation, sexual evolution, and breeding. Compared to available medaka genomes, *O. curvinotus* is larger than *O. latipes* but similar to *O. javanicus*, facilitating comparative genomics (NCBI Genome Data 2022). Overall, this reference enables crucial biological studies to inform conservation efforts for *O. curvinotus*.

As *O. curvinotus* inhabits complex, rapidly‐changing mangrove waters, its genome shows signatures of adaptation. *O. curvinotus* had over 200 species‐specific genes compared to related medakas (Figure 2a), suggesting genomic innovations. Numerous expanded gene families were enriched for metabolic pathways involving *CYPs* (Figure 3b,c), which are important for responding to variable mangrove environments and pollutants (Nebert et al., 1991).

Moreover, we identified 69 *O. curvinotus CYPs* (*OcuCYPs*) spanning 18 families and 10 clans, illuminating the *CYP* superfamily in this species (Table S8). Compared to known vertebrate *CYPs*, *O. curvinotus* lacks the *CYP39* family found only in zebrafish among fish (Nelson et al., 2013). Intriguingly, *O. curvinotus* possesses *CYP16*, unlike most teleosts (Dermauw et al., 2020). The function of this newly described family in vertebrates remains unknown (Nelson, 2011). Among Oryzias, only the closely‐related *O. latipes* also contains *CYP16* (Zhang et al., 2014).

Furthermore, numerous *OcuCYPs* exhibited tandem duplications, mirroring the comparative genomic expansions (Figure 3a). Such duplication commonly drives *CYP* diversification, likely adapting metabolite regulation (Baldwin et al., 2009). For instance, the expanded *CYP2* and *CYP3* families metabolize xenobiotics and synthesize regulatory compounds (Kashiwada et al., 2005; Lee et al., 2001). The responsiveness of multiple *OcuCYPs*, including *CYP1A*, *CYP2N18/19*, and *CYP2Y30*, to model pollutants demonstrates their expanded importance in detoxification (Figure 6a).

Quantitative data on many expanded *OcuCYPs* showed liver‐elevated expression, fitting known *CYP* detoxification roles (Uno et al., 2012). Gill/skin expression also enables initial toxin processing before hepatic metabolism. Together, the specialized tissue distribution and pollution responsiveness of expanded *OcuCYPs* facilitates rapid defense against fluctuating mangrove contaminants. Also, RNA‐seq revealed diverse *OcuCYP* expression during *O. curvinotus* development (Figure 5a). Continuous *CYP11A2* expression may regulate migration in embryogenesis (Hsu et al., 2006). Overall, variable developmental patterns suggest *OcuCYP* roles in responding to mangrove dynamics and regulating physiology.

Sex‐biased gonadal *CYP* expression points to reproductive functions (Figure 5c,d,e,f). *CYP19* subtypes showed distinct gender‐enriched expression fitting known roles in zebrafish – *CYP19A1* ovary‐biased and *CYP19A2* female‐dominant in brains (Kishida & Callard, 2001; Wang & Ge, 2004). *CYP17*, involved in sex steroid synthesis, also exhibited testis‐elevated expression, implying significance in sex reversal (Yu et al., 2003).

While this study characterized *OcuCYPs*, key questions remain on their developmental, reproductive, and ecotoxicological functions. Assessing pollutant impacts during *O. curvinotus* embryogenesis could reveal effects on growth and sex differentiation. Investigating *CYP* substrates and regulation will also refine understanding of their diverse roles enabling survival in changing mangrove environments.

## AUTHOR CONTRIBUTIONS

**Ming Li:** Data curation (equal); formal analysis (equal); investigation (equal); methodology (equal); project administration (equal); resources (lead); software (lead); supervision (lead); validation (lead); visualization (lead); writing – original draft (lead); writing – review and editing (lead). **Aiping Deng:** Data curation (equal); formal analysis (equal); investigation (equal); methodology (equal); project administration (equal); resources (lead); software (lead); supervision (lead); validation (lead); visualization (lead); writing – original draft (lead); writing – review and editing (lead). **Chuanmeng He:** Data curation (equal); formal analysis (equal); investigation (equal); methodology (equal); project administration (equal); resources (supporting); software (supporting); supervision (supporting); validation (supporting); visualization (supporting); writing – original draft (supporting); writing – review and editing (supporting). **Zebin Yao:** Data curation (equal); formal analysis (equal); investigation (equal); methodology (equal); project administration (equal); resources (supporting); software (supporting); supervision (supporting); validation (supporting); visualization (supporting); writing – original draft (supporting); writing – review and editing (supporting). **Zixuan Zhuo:** Data curation (equal); formal analysis (equal); investigation (equal); methodology (equal); project administration (equal); resources (supporting); software (supporting); supervision (supporting); validation (supporting); visualization (supporting); writing – original draft (supporting); writing – review and editing (supporting). **Xiu yue Wang:** Data curation (equal); formal analysis (equal); investigation (equal); methodology (equal); project administration (equal); resources (supporting); software (supporting); supervision (supporting); validation (supporting); visualization (supporting); writing – original draft (supporting); writing – review and editing (supporting). **Zhongduo Wang:** Conceptualization (lead); data curation (lead); formal analysis (lead); funding acquisition (lead); investigation (lead); methodology (lead); project administration (lead); supervision (lead).

## CONFLICT OF INTEREST STATEMENT

The authors declare that they have no competing interests.

## CODE AVAILABILITY

All data analyses were performed according to the manual and protocols of the published bioinformatic tools. The version and parameters of software have been described in Methods section.

## INSTITUTIONAL REVIEW BOARD

All experimental protocols in this study were approved by the Animal Research and Ethics Committee of Guangdong Ocean University Zhanjiang, Guangdong, China (201903003).

## Supporting information


Table S1

Table S2

Table S3

Table S4

Table S5

Table S6

Table S7

Table S8

Table S9

## Data Availability

Raw sequencing reads have been made publicly available through the NCBI Sequence Read Archive (PRJNA1033149). Structural and functional annotations of *Oryzias curvinotus* can be found in figshare public repository (https://doi.org/10.6084/m9.figshare.24524854.v1). The genome sequences are available in the NCBI Sequence Read Archive (PRJNA821560).
